# Respiratory Syncytial Virus Infection: Treatments and Clinical Management

**DOI:** 10.3390/vaccines11020491

**Published:** 2023-02-20

**Authors:** Shiza Malik, Tahir Ahmad, Khalid Muhammad, Yasir Waheed

**Affiliations:** 1 Bridging Health Foundation, Rawalpindi 46000, Pakistan; 2 Industrial Biotechnology, Atta ur Rahman School of Applied Biosciences, National University of Sciences and Technology, Islamabad 44000, Pakistan; 3 Department of Biology, College of Science, UAE University, Al Ain 15551, United Arab Emirates; 4 Office of Research, Innovation, and Commercialization (ORIC), Shaheed Zulfiqar Ali Bhutto Medical University, Islamabad 44000, Pakistan; 5 Gilbert and Rose-Marie Chagoury School of Medicine, Lebanese American University, Byblos 1401, Lebanon

**Keywords:** respiratory syncytial virus, RSV infection, therapeutics, antiviral agents, vaccines, therapies, treatment, novel therapeutic, clinical management

## Abstract

Respiratory syncytial virus (RSV) is a major healthcare concern, especially for immune-compromised individuals and infants below 5 years of age. Worldwide, it is known to be associated with incidences of morbidity and mortality in infants. Despite the seriousness of the issue and continuous rigorous scientific efforts, no approved vaccine or available drug is fully effective against RSV. The purpose of this review article is to provide insights into the past and ongoing efforts for securing effective vaccines and therapeutics against RSV. The readers will be able to confer the mechanism of existing therapies and the loopholes that need to be overcome for future therapeutic development against RSV. A methodological approach was applied to collect the latest data and updated results regarding therapeutics and vaccine development against RSV. We outline the latest throughput vaccination technologies and prophylactic development efforts linked with RSV. A range of vaccination approaches with the already available vaccine (with limited use) and those undergoing trials are included. Moreover, important drug regimens used alone or in conjugation with adjuvants or vaccines are also briefly discussed. After reading this article, the audience will be able to understand the current standing of clinical management in the form of the vaccine, prophylactic, and therapeutic candidates against RSV. An understanding of the biological behavior acting as a reason behind the lack of effective therapeutics against RSV will also be developed. The literature indicates a need to overcome the limitations attached to RSV clinical management, drugs, and vaccine development that could be explained by dealing with the challenges of current study designs with continuous improvement and further work and approval on novel therapeutic applications.

## 1. Introduction

Historically, respiratory syncytial virus (RSV) was discovered in 1956 as the causative agent of lower-respiratory-tract infections (LRTI) in children worldwide [1]. It also causes infection in elderly and immune-compromised individuals. This is unlike in pediatric patients, for which understanding is well developed [2]. There is a general lack of understanding of RSV infection and transmission routes in elderly patients. It should be noted that RSV majorly causes infection in babies in forms of inflammation (pneumonia) or bronchiolitis (inflammation of the lungs and airways). The infection develops in the form of a runny nose, low fever, sore throat, dry cough, sneezing, headache, and breathing difficulty. Hospitalization and death cases have also been recorded worldwide, especially in infants. Thus, there is urgent need to call for immediate therapeutic development against RSV [2]. However, the vulnerable nature of an infant’s respiratory and immune system makes it difficult to develop effective vaccine candidates due to limited testability in children. The past vaccination trials of mid 20th century and the resultant hospitalization cases have also restricted the clinical research trials [3]. This aspect is further discussed later in this article.

Biologically, RSV belongs to the family Paramyxoviridae of viruses, characterized by a single-stranded RNA genome with a length of approximately 15 kB [3,4]. This genome accounts for nearly 10 genes and 11 protein entities that involve surface glycoproteins and those involved in the fusion (F) and attachment (G) of the virus, among others [5,6,7]. These proteins also serve the purpose of antigenic targets for therapeutic and vaccine development. The relative antigenic differences among these proteins, especially glycoproteins, have resulted in the deviation of RSV into two subgroups (A and B) [8,9,10]. In terms of physical characteristics, the RSV exists in spherical or long filamentous forms [11]. According to the reports of the WHO, RSV is responsible for a yearly ~39 million infection cases worldwide. Some of these infections reportedly lead to hospitalization (~3.6 million) and severe morbidity cases that lead to ~101,400 deaths per year [12]. This case is even more severe for developing countries with compromised healthcare and economic conditions. In these countries, the communities are considered as vulnerable to RSV as they are to influenza virus incidences, which is also quite common there [12,13,14].

Immunologically, the main features that make infants and children vulnerable to RSV infection may include an overall immunocompromised system that arises due to conditions such as premature birth conditions, age (≤6 months–1 year), bronchopulmonary dysplasia, neuromuscular disorders, and compromised congenital heart and lung conditions at birth [13,15,16]. Moreover, the risk factors also involve the male gender, the incidence of trisomy 21, and cystic fibrosis upon birth [3,12,17]. Clinically, RSV infection exhibits the features of sore throat, malaise, and coryza, in mild incidences [18]. However, in the case of the increased infectious form of LRTI, the clinical outcomes include low-grade fever, wheezing, dyspnea, cyanosis, and subcostal recession with consolidation. It causes 20% of pneumonia cases in infants [19,20,21].

The current treatment options are in the form of supportive and preventive options that are used for RSV-associated infant health management worldwide [21]. To date, no effective or approved therapeutics and vaccines are available to cure this virus disease [12]. Only short-time clinical benefits are achieved with the use of the available drugs and antivirals or with the hormonal treatment option (nebulized adrenalin), with little or no effect from chemotherapy based on hypertonic saline, a bronchodilator, glucocorticoid, etc. [3,11,14,22,23]. Several efforts are being put forth in the field of vaccinology and pharmaceutical for RSV clinical management. The preclinical and early clinical outcomes of these studies present hope that the future might hold the approval chances of an effective therapy option against RSV [24,25,26,27].

There exists massive information on RSV virology related to its replication, transmission, and pathogenesis, which enables therapeutic design against RSV infection. However, there still exists a gap in standardized global surveillance, diagnosis, and data management regarding RSV [6,28,29]. Moreover, there is a general inefficiency in prophylaxis and antiviral and vaccination strategies against RSV. This review revises our understanding of the general pathophysiological features of RSV infection and the experimental default lines that exist for therapeutic development against it [30,31]. Moreover, we mainly discuss different vaccination and therapeutic developments in the domain of RSV clinical management and the understanding of the lack of market approval for these tested drugs [32,33,34,35,36,37,38,39]. After reading this article, the audience will be able to understand the gaps in the literature and clinical developments that need to be undertaken via integrated and collaborative clinical research efforts so that RSV and associated infections can be well managed in the next couple of years.

## 2. Main Body

### 2.1. The Ever-Evolving and Mutational Nature of RSV

RSV types A and B are the causative agents of infectious human respiratory syncytial virus infection. The internal structural proteins of RSV consist of matrix protein (M) and nucleoprotein (N), which contribute to the functions of the polymerase complex consisting of phosphoprotein (P) and polymerase (L) [40,41,42,43,44]. Moreover, nonstructural proteins such as surface glycoproteins (G), small hydrophobic protein (SH), fusion protein (F), and the regulatory M2 proteins function to regulate the innate immune responses by acting as antigenic particles within the host body [45,46]. They also function in the transcription and replication of virus particles. The overall RNA-dependent replication cycle is often faced with an error in the replication and proofreading mechanism [9,47].

Certain mutations are exhibited in the form of single-nucleotide polymorphisms (SNPs) and other point mutations that cause changes in virus virulence and, hence, the subsequent immune responses in the host [48]. Moreover, the changes also affect the therapeutic efficiency of antiviral agents that are ineffective against the specific viral genetic makeup [32]. These characteristics make it pertinent for the scientific community to offer a more integrated approach to designing a combination therapy involving the co-application of certain vaccines, drugs, and adjuvants with a prime boosting effect so that a universal therapy can be devised for the clinical management of future RSV infections [3,13,14].

### 2.2. Updates on Vaccine Development against RSV

As of now, no specific RSV vaccines are licensed or approved for use at the worldwide level [12]. However, it is important to formulate a proper vaccine against RSV infections since the target group involves immune-compromised individuals that have higher chances of infection development; these may involve infants and children, the elderly, and pregnant women [17]. If the infected cases remain untreated, they are likely to increase mortality and hospitalization counts, thus creating a great burden on the healthcare system worldwide [49]. The failed experiments on inactivated and subunit vaccine formulations such as the formalin-inactivated alum-adjuvanted RSV vaccine (FI-RSV) have caused a stoppage or delay of further vaccination trials in infants owing to the clinical outcomes that resulted upon their application in children [11]. Scientists are, thus, sorting out ways to create better immune regulatory substances and therapeutic options that can manage the illness symptoms without causing any further increase in symptoms [19].

An ideal vaccine candidate should be able to induce immune responses rapidly and should be administered within the first month of birth to avoid infection that develops in the period ranging from the sixth week to the sixth month [19,20]. However, the major cause of delay in vaccine development and its clinical implication is the immunocompromised nature of infants and the presence of antibodies acquired from the mother, which delays and reduces the vaccine responses [21,50]. Since active vaccination is hurdled due to the passive immunization from the mother, the replicating and vector-based vaccines are the preferred choices for testing in infants rather than non-replication vaccines [51,52]. In addition to these hurdles, several pieces of evidence validate the inferences about the development of effective vaccines against RSV. One of these implications is the chances of development of severe infection even with the chance of RSV infection development throughout life [20,53,54]. Secondly, the monoclonal antibody derived from palivizumab is effective against increased mortality and morbidity cases. Yet another implication comes from the correlation between the maternal antibody-based neutralization effect that offers immune responses to a certain degree and helps protect against hospitalization [20,54].

More specifically, the F and G proteins of RSV induce the induction of neutralizing antibody-based protective effects [55]. Thus, they often choose to design vaccines for cross-protective RSV strain responses. The F protein is comparatively even better at immune induction than the G protein and is more conserved among varying RSV strains compared to the G protein, which is conserved only at a few central protein sequences [56]. These passive and active antibody-mediated immune responses offer a preventive capacity against RSV disease. These vaccines are designed to avoid problems associated with preexisting immunity in infants from mothers and excessive immune evasion or elevation that could be problematic for immunocompromised vulnerable patients [29].

Several vaccination strategies have been evaluated recently such as live-attenuated, recombinant vector-based, peptide-based, and subunit vaccines, which are briefly discussed in the next section; examples of a few candidates under trial are presented in Table 1. Despite the increase in ongoing RSV vaccination trials, the investigation is mostly limited to phases 1 and 2a of clinical experimentation because several challenges remain [18,50,57]. A few causes are ethical concerns, the immunocompromised nature of patients, inefficient surveillance rates, and lack of research funding in the prevalent healthcare systems [27,58,59].

Moreover, the useful profiles of RSV vaccines have also demonstrated limited clinical effectiveness in humans. Nonetheless, even though the vaccine candidates designed under various methods have exhibited good immunogenic profile in animal models, their impact in humans has largely remained ineffective, incompetent, and inclusive [40,60,61,62,63]. Therefore, more effective vaccination trials are needed that may originate from different vaccination approaches coupled with widescale clinical experimentation on both animal and human subjects for improved vaccination protocols and their widescale applicability and approval. Some major vaccination approaches in the research lane for RSV management are briefly discussed next.

**Table 1:** Vaccines against RSV under clinical trials.

Sr. No.	Vaccine Strategy	Candidates under Trial	References
1.	Live-attenuated vaccines against RSV		
A.	Vaccine candidates based on(M2-2 gene deletion) (M2-2 regulates RNA replication)	LID ΔM2-2 vaccine LID ΔM2-2 1030s LID cp ΔM2-2 MEDI ΔM2-2 MEDI-559	[64,65,66,67,68]
B.	Vaccine candidates based on SH gene deletion( SH gene regulates cell apoptosis and role in viral fusion )	The rA2cp248/404/1030ΔSH vaccine The MEDI–599 vaccine OE4 (RSV-A2-dNS1-dNS2- ΔSH-dGm-Gsnull-line19F) DB1 (RSV-A2-dNS-ΔSH-BAF), RSV cps2 vaccine	[23,24,25,26,27,28,29,30,64]
C.	Vaccine candidates based on NS2 gene deletion(NS2 gene regulates epithelial cell shedding and host IFN based immune responses)	ΔNS2/Δ1313/1314L or MEDI-559	[67,68,69,70]
D.	Vaccines based on genes from RSV related viruses	MEDI–534 vaccine (parainfluenza virus type 3 (PIV3) genome engineered to express RSV F protein)	[64,65,66,67,68,69,70]
E.	Other vaccine candidates	*cpts*-248/404 vaccine	[64,65,66,67,68,70]
2.	Vector-based vaccines		
A.	Adenovirus vector-based vaccine trials	GSK3003891A VXA-RSV-f Ad26.RSV.FA2 Ad35.RSV.FA2 RSV 001 ChAd155-RSV (GSK3389245A)	[3,9,64,65,66,67,68,69,70]
B.	PIV3 vector	MEDI–534	[17,18,22,28],
C.	Adenovirus vector and an MVA vector	MVA-RSV MVA-BN PanAd3-RSV and	[17,18,29,57],
D.	RSV vaccine designed on Simian adenovirus	PanAd3-RSV) (prime boost strategy)	[13,14],
3.	Recombinant live-attenuated RSV	RSV LID ΔM2-2 RSV cps2, Lot RSV#005A	[64,70]
4.	Protein and nanoparticle-based vaccines	RSV sF antigen + synthetic glucopyranosyl lipid A adjuvant F-protein nanoparticle + aluminum hydroxide adjuvant MEDI-7510 (Post-fusion F glycoprotein + glucopyranosyl lipid A adjuvant)	[66,69,70,71,72,73,74,75,76,77,78]
5.	Subunit vaccines against RSV	MEDI 7510 (RSV sF antigen + synthetic TLR-4 agonist /with or without synthetic glucopyranosyl lipid A adjuvant) RSV F subunit vaccine GSK3003891A (Prefusion F subunit vaccine) Novavax’s RSV (F-protein nanoparticle vaccine (with and without an aluminum hydroxide adjuvant)	[7,53,70,73,74]

#### 2.2.1. Live-Attenuated Vaccines against RSV

The failure of inactivated vaccine formulations in the 1960s led to experiments in live-attenuated vaccine designs. These vaccines are prepared by rendering the virus ineffective by treating it with chemicals, radiation, or extreme temperatures [64]. Moreover, the virus may be subjected to genetic deletions for the maintenance of only a limited tendency of the virus genome for replication and antigenic representation in hosts [65]. A well-known procedure involves the deletion of a large segment of the M2-2 gene that plays a vital role in RNA replication. On one hand, it decreases the viral replication; in contrast, it causes increased expression of F and G proteins that act as antigenic representations and make the virus adequately attenuated for immune responses in the form of the production of neutralizing antibodies [66,67,68]. The risk of de-attenuation is kept low to avoid associated harmful immunological responses.

These designs are effective in terms of lower chances of immunological exacerbation upon future RSV exposure in infants and ease of administration, which limits the viral infection to the upper respiratory tract [65,66]. In terms of a systematic response, they induce mucosal immunity and humoral immune responses coupled with the passive immunity acquired by the mother. The main advantages associated with live-attenuated vaccination protocol are the easier intranasal administration route, the induction of first-hand immune responses, and the lower chances of exacerbated immune responses against pre-exposure to RSV [64,65]. A disadvantage may include the need to acquire a balance between immunogenicity and attenuation adequacy [64]. Trials are underway to reduce the high rates of antibody titers in infants. This approach has been in clinical trials for a long time; some examples of ongoing clinical trials on live-attenuated vaccines were provided in Table 1.

#### 2.2.2. Vector Delivery Systems against RSV

In this approach, some nonpathogenic virus vectors are used to express RSV proteinaceous candidates, such as the adenovirus vector system, in which portions of the RSV virus genome, such as F, N, G, and M2-1 proteins, are expressed [13,14]. These vectors help modulate immunostimulatory functions in host bodies. These protein entities regulate T-cell-mediated responses by expressing the epitopes. They limit the cellular immune responses without causing insufficient viral attenuation [69,70]. Moreover, these vectors allow the adjuvant application to regulate better immunostimulatory profile management in clinical experiments. The virus vectors most used include adenovirus and modified vaccinia Ankara (MVA) [63].

A major advantage of a vector-based system is the induction of potent cellular and humoral response hosts and better safety profiles with no risk of insufficient attenuation as compared to live-attenuated vaccines, particularly in infants [1]. However, the concern about the required prior exposure to the vector and immunological memory may limit the immunostimulatory effect [31]. Moreover, the potential oncogenicity and pathogenicity associated with some virus vectors may also be major hurdles to be overcome for effective vaccine development. Vaccine trials based on these vector systems have exhibited moderate to self-limiting immunogenic responses at the site of injection with an overall efficacy in infants. However, further clinical applications are required for licensure [31,71,72].

#### 2.2.3. Protein/Peptide or Subunit Vaccines

In addition to live-attenuated and vector vaccine formulations, the protein or peptide or subunit vaccination approaches are effective vaccination candidates for RSV management [73]. Protein/peptide-based vaccines function like the RSV-neutralizing antibodies that are transferred passively from mother to fetus during pregnancy and, thus, cause the development of passive immunity in infants [74,75]. Protein-based vaccines involve the use of whole viruses that are rendered inactivated or without the subunit antigens and viral particles [75]. These candidates are designed with different sizes and amounts of adjuvants to allow the partial release of viral particles over time [74]. One of the major advantages of peptide or protein-based vaccines is the effectiveness associated with material immunization via transplacental antibody transfer, which is beneficial for infants. However, the high risk of immune-stimulatory exacerbation may be a threat to infant application [73,74]

#### 2.2.4. mRNA Vaccines against RSV

Nucleic acid-based vaccines on mRNA and DNA have been in practice for many years. mRNA, however, is not preferably used as a tool of vaccine design due to the associated concerns of instability, poor efficacy, and potential to stimulate an excessive immune response, which is not needed for vaccination of RSV-infected individuals [76,77]. These vaccines are transferred subcutaneously to the skin and muscle surfaces where they act via an antigenic expression system to deliver epitopes to be displayed T and B cells of the immune system [59,78]. Owing to the chance of exclusive immune responses, the current mRNA-based trials are mostly limited to adults and the elderly, and they require further work to establish efficacy in infants and children.

### 2.3. Immuno-Prophylaxis for RSV Management

Prophylaxis pertains to the immunomodulating, regulating, and stimulatory preparations that are developed to induce active and passive immunity generation in hosts [50,51]. Certain RSV immunoglobulin preparations are carried out which contain monoclonal and polyclonal antibodies derived from donors that exhibit high RSV-neutralizing antibody titers [27,50]. These immunoprophylactic reduce the overall mortality and hospitalization rates in infants [27]. However, there are some issues associated with these immunologic preparations such as the need for long-term infusion and hospitalized supervision, high column dosages with the potential risk of excessive immune responses, and bloodborne pathogen development [54,79]. Similarly, they often make it impossible to apply other birth-associated vaccination drives such as against measles/mumps/rubella (MMR) vaccine or the smallpox vaccine [20,21,80]. Moreover, the issues of high cost and difficulty of manufacturing are also some issues challenges that need to be overcome for more profound immunoregulatory prophylaxis against RSV infection [20,21].

### 2.4. Molecular Insights into the MOA of Antivirals against RSV

Antiviral therapies are quite a known field with almost similar technicalities and processes for different virus infections [81]. The drug development processes are quite similar and well acquainted. Moreover, the technical barriers associated with these processes have been systematically reduced with the upgradation of biotechnologies and bioinformatics tools. The well-known platforms of bioinformatics and in silico modules are now increasingly used for in vitro modeling and clinical experimentation [81,82]. However, clinical experimentation and human-based models are still challenged owing to ethical health-related concerns. However, the available literature regarding the preclinical and early clinical data is immense for viral targets and holds hope for developing suitable antiviral candidates against RSV in the future [57,83].

The replication mechanism of RSV and its linked biological traits have been well studied, although gaps still exist regarding the exact nature of the infection. However, the available information is immense to design relevant and effective antiviral agents [40]. As elaborated earlier, the RSV genome encodes ~11 proteins, among which three majorly contribute to the viral envelope structure, namely, the glycoprotein (G), fusion (F), and small hydrophobic (SH) [45]. All these are utilized for the development of vaccine candidates (as explained earlier). Other proteins such as M2–1, M2-2, NS1, and NS2 are involved directly or indirectly in the viral replication procedure [45,46]. The similar and conserved nature of some of these proteins suggests that antiviral agents could be manufactured with inhibitory potential across different virus species.

Similarly, the different surface characteristics of the host cell such as cell surface receptors and binding domains can also be utilized to design antiviral agents in the case of RSV [9,23]. For example, nucleolin is a receptor specific for RSV F protein recognition. It acts as a molecular shuttle to help move material across intracellular compartments. Thus, certain drugs are in trial phases that are repurposed for binding nucleolin (examples are provided in Table 2) [78]. Similarly, the various steps in virus replication and the viral particles involved in those steps can be potentiated as targets for antiviral designs. Factors such as glycosaminoglycans, the fractalkine receptor, RhoA, cell surface CX3CR1, and the aforementioned nucleolin, all of which contribute to viral attachment, entry, and replication steps, are good targets for antiviral therapies [78,84,85].

Yet another approach is the use of Synthetic RNAi derivatives such as short RNA interference (siRNA) that function to specifically target and degrade mRNA function and deregulate the expression of virally encoded proteins [86,87]. RNAi-based therapeutics have already shown efficacy in several other infections of genetic, cancerous, and viral origins [22,88]. Moreover, the inhibition of inflammatory agents in the host cell and regular inflammatory immune responses upon RSV infection are other effective mechanisms to control RSV pathogenesis. By targeting specific agents such as neutrophils (responsible for severe RSV-associated lung infection) an effective antiviral therapy can be proposed [22,87].

Additionally, targeting the virus surface molecules, such as F protein or G protein, could be an effective approach for the pharmacological inhibition of virus infection [11]. Some of the prophylaxis immunoglobulins are used to utilize these surface proteins, present them on antigen-presenting cells, and regulate immune responses through neutralizing antibody production [9,89]. Similar mechanisms are also used for vaccine development, as previously elaborated. Another important mechanism of antiviral development is to formulate antiviral agents that interfere with polymerase activity and, hence, avert virus replication and infection [47]. Such methods are accounted for as viral gene silencing. Moreover, by targeting the chemokines receptors such as CX3C and other related receptors, the RSV-induced pathological and inflammatory responses could be averted [22,90]. Furthermore, the combined application of host-directed inhibiting agents and antiviral agents is another attractive approach for designing effective therapeutics, but there is a need to keep an account of cellular toxicity [11,13].

Most of the described antiviral approaches involve the consideration of viral loads. However, there is conflicting inference as to whether the viral load correlates with the clinical severity of RSV infecting humans or not [17,18,29,91]. This is because different studies have devised different inferences. These data suggest that a combinational approach of antiviral and anti-inflammatory agents with immune-regulatory prophylaxis-based treatment could be better against overall pathogenesis. A combined effect will regulate viral and host factors, as well as immune responses in humans. Therefore, future viral therapies should concentrate more on combination, prime boosting, and co-application approaches [18,19,20,50,54,92]. Some important antiviral strategies, along with particular examples and clinical trials, are explained briefly in Table 2.

**Table 2:** Antiviral agents and therapeutics in clinical development against RSV.

Sr. No.	Antiviral and Therapeutics Approach	Candidates under Clinical Trials	References
1.	Viral cellular entity inhibitors	Aptamers (nucleolin-binding agents) (G)-rich oligonucleotide (nucleolin inhibitor)	[5,6,30,31,56,62,63,80,93,94,95,96,97,98,99,100,101,102]
2.	Host virus fusion inhibitors	GS-5806 (presatovir) VP-14637 (renamed to MDT-637and JNJ-2408068) ALX-0171	[9,11,23,29,48,70,100,103,104,105,106,107]
3.	Polymerase inhibitors	ALS-8176 T-705 (favipiravir) RSV-604 (A-60444)	[22,47,68,108,109,110,111,112,113]
4.	Nucleoside analog inhibitors	Ribavirin (NA inhibitor of RNA synthesis) ALS-008176	[9,11,22,23,47],
5.	Synthetic RNAi derivatives	ALN-RSV01(siRNA against the RSV nucleocapsid (N) protein) ALS-008176 (NA)	[49,88,106,114,115,116],
6.	Targeting inflammatory molecules/anti-inflammatory molecules	Danirixin (GSK1325756) RV568, (narrow spectrum kinase inhibitor)	[20,21,27,50,51,54,58,79],
7.	Prophylactics and immunoregulators	MEDI 557 (recombinant human monoclonal antibody) MEDI 8897 (human RSV monoclonal antibody) RI-001 (polyclonal antibody) Motavizumab (MEDI-524), derived from palivizumab Trivalent nanobody ALX-0171 D25 RSV antibody (against F glycoproteins) (RSV-IVIG, RespiGam) intravenous immunoglobulin Palivizumab (humanized monoclonal IgG1 antibody) ALX-0171 (a nanobody preparation) REGN2222, (human monoclonal IgG1 antibody against RSV-F) MEDI-557 (derived from motavizumab) MEDI-8897, derived from D25-, an anti-RSV F MAb)	[106,117,118,119,120,121,122,123,124,125,126,127,128]

### 2.5. RSV Infection, Vulnerability of Infants, and the Vaccination Efforts

As elaborated earlier, RSV majorly affects infants; thus, the major hurdle in RSV vaccine development is the vulnerable nature of the immune system in infants and children [129]. Vaccination efforts to prevent RSV infection in infants have mainly resulted in kids becoming sick, according to recent research [130]. These findings have also helped provide essential clues regarding safe and effective vaccines and therapeutic development against RSV [130]. The earlier vaccination trials that date back to the 1960s confined future vaccination campaigns owing to the hospitalization cases that arose in vaccinated children [129]. In those trials, inactivated virus particle-based vaccines were utilized. Approximately 8% of children became hospitalized following vaccination, and two died of infection [128,129,130].

It remains unclear whether improper inactivation or the chemical formalin (used for inactivation) resulted in morbidities in those children. The scientific rigor in later years helped to overcome this confusion [130]. Researchers provided information about why the vaccine caused illnesses such as enhanced respiratory disease (ERD). The resulting causes indicated that the children's immune system (antibodies) was not binding strongly enough with the inactivated virus particles (antigens) to produce effective and protective immune responses [128,129,130]. The antibodies were found to be dragging the dead viral particles with them, which resulted in triggered immune responses from other cells of the immune system in those patients [129]. A better vaccine candidate or therapeutic agent (based on attenuated viruses) may be required in the future to trigger stronger immune responses [100,101,102]. Thus, there is a need to follow appropriate procedures and coordinate clinical research trials to develop successful therapeutics and vaccines against RSV infection in the coming years [130].

## 3. Conclusions

Respiratory syncytial virus (RSV) remains a pathogenic threat to immunocompromised patients worldwide. Despite enormous insight into RSV pathology, some data regarding the pathogenesis remain unaddressed, thus necessitating further exploration for future antiviral development. Only a limited number of drugs or vaccines have been approved against RSV, and they also exhibit some limitations for global clinical deployment unlike other viral infections and therapeutic developments. Limited data are available regarding specific therapeutics and licensed vaccines for controlling the associated healthcare dilemma. Most of these trials are stuck in clinical phase 1 or 2; thus, further clinical practices are necessary for licensure and approval. The traditional strategies of vaccines and antiviral agents are in the redevelopment and upgradation phase. Importance should also be directed toward novel approaches such as those involving nanotechnology, immunomodulators, molecular inhibitors, gene silencers, and siRNA. Researchers will require a profoundly integrated and well-knitted effort, involving bioinformaticians and pharmacologists, to propose an effective understanding of RSV pathogenesis and clinical practice of novel vaccines, prophylactics, and antiviral therapeutics. The role of healthcare authorities such as the FDA, WHO, and CDC will also remain important for figuring out ways for improvising clinical experiments through the provision of funding, as well as appropriate recommendations for the community-level application of approved and licensed drugs. Moreover, industrial involvement, community acceptance, and wide-spectrum application are some other challenges that remain to be effective and immediately dealt with to prevent further deaths from RSV in the future.

## Data Availability

Not applicable.

## Abbreviations

CDC	Centers for Disease Control and Prevention
CXC	C chemokine family
F	fusion protein
FDA	Food and Drug Administration
G	glycoprotein
L	polymerase
LAV	live-attenuated vaccine
LRTI	lower-respiratory-tract infection
M	matrix protein
M2	regulatory protein
MAbs	monoclonal antibodies
MVA	modified vaccinia Ankara
N	nucleoprotein
P	phosphoprotein
RhoA	Ras homolog family member A
RNAi	RNA interference
RSV	respiratory syncytial virus
SH	small hydrophobic protein
SNPs	single-nucleotide polymorphisms
VLPs	virus-like particles
WHO	World Health Organization
